# Crowd-sourcing observations of volcanic eruptions during the 2021 Fagradalsfjall and Cumbre Vieja events

**DOI:** 10.1038/s41467-022-30333-4

**Published:** 2022-05-11

**Authors:** Fabian B. Wadsworth, Edward W. Llewellin, Jamie I. Farquharson, Janina K. Gillies, Ariane Loisel, Léon Frey, Evgenia Ilyinskaya, Thor Thordarson, Samantha Tramontano, Einat Lev, Matthew J. Pankhurst, Alejandro Galdeano Rull, María Asensio-Ramos, Nemesio M. Pérez, Pedro A. Hernández, David Calvo, M. Carmen Solana, Ulrich Kueppers, Alejandro Polo Santabárbara

**Affiliations:** 1 Earth Sciences, Durham University, Durham, DH1 3LE UK; 2 Independent, Edinburgh, UK; 3 focusTerra, Department of Earth Sciences, ETH Zurich, Zurich, Switzerland; 4 COMET, School of Earth & Environment, University of Leeds, Leeds, UK; 5 Faculty of Earth Sciences, University of Iceland, Sturlugata 7, 101, Reykjavík, Iceland; 6 The Graduate Center, CUNY, 365 Fifth Avenue, New York, NY 10016 USA; 7 Lamont-Doherty Earth Observatory, Columbia University, New York, NY USA; 8 Instituto Volcanológico de Canarias (INVOLCAN), La Laguna, Santa Cruz de Tenerife Spain; 9 Overon Aerial, Av Isaac Newton 8, mediapro building, Getafe, 28906 Madrid, Spain; 10 School of the Environment, Geography, and Geosciences, University of Portsmouth, Portsmouth, PO1 3QL UK; 11 Earth and Environmental Sciences, Ludwig-Maximilians-Universität, Theresienstr. 41, 80333 München, Germany; 12 Datadron S.L. C/San Agustín, 72 Los Realejos, 38410 Santa Cruz de Tenerife, Tenerife Spain

**Keywords:** Natural hazards, Environmental impact, Research data

## Abstract

This study explores the scientific potential of crowdsourced observations during volcanic eruptions, using the 2021 Fagradalsfjall (Iceland) and Cumbre Vieja (Canary Islands) events as case studies.

## Teaser

Two remarkable 2021 eruptions – Fagradalsfjall (Reykjanes, Iceland) and Cumbre Vieja (La Palma, Spain) – have been captured in imagery and videography at unprecedented spatial and temporal resolution by tourists, volcano-enthusiasts, and media organizations. We propose this represents the start of a new era for observational volcanology, with the abundance of eruption imagery leveraged for scientific use.

The Fagradalsfjall^1^ and Cumbre Vieja^2–4^ eruptions were spectacular and, in the case of Cumbre Vieja, highly destructive. Over their course, the eruptions waxed and waned, fed lava flows, caused numerous felt earthquakes and explosions, localized from crack-fed fissure eruptions into discrete vents and cones, formed fountains up to hundreds of meters high^5–7^ and, for Cumbre Vieja, sent buoyant plumes of pyroclasts and gas to high altitudes. Importantly, both eruptions were visited by people from all over the world and broadcasted widely. While those visitors included many volcanologists, the majority were tourists and volcano enthusiasts, and included photographers, videographers, artists, content creators, and drone pilots.

Just 5 years ago, the use of drones on volcanoes was the preserve of a few researchers only^8^. Today, drones are a mainstay of the photographer’s or videographer’s arsenal, whether amateur or professional. Thanks to the rapid increase in quality and availability of high-resolution digital photography equipment, and the increasing quality of smartphone cameras, it is possible for the enthusiastic amateur to capture natural phenomena in unprecedented detail. Here, we compare and contrast the Fagradalsfjall and Cumbre Vieja eruptions through the lens of the content creators who have visited these sites, with the aim of assessing the utility of non-specialist content for scientific purposes.

## Fagradalsfjall

**Fig. 1:**
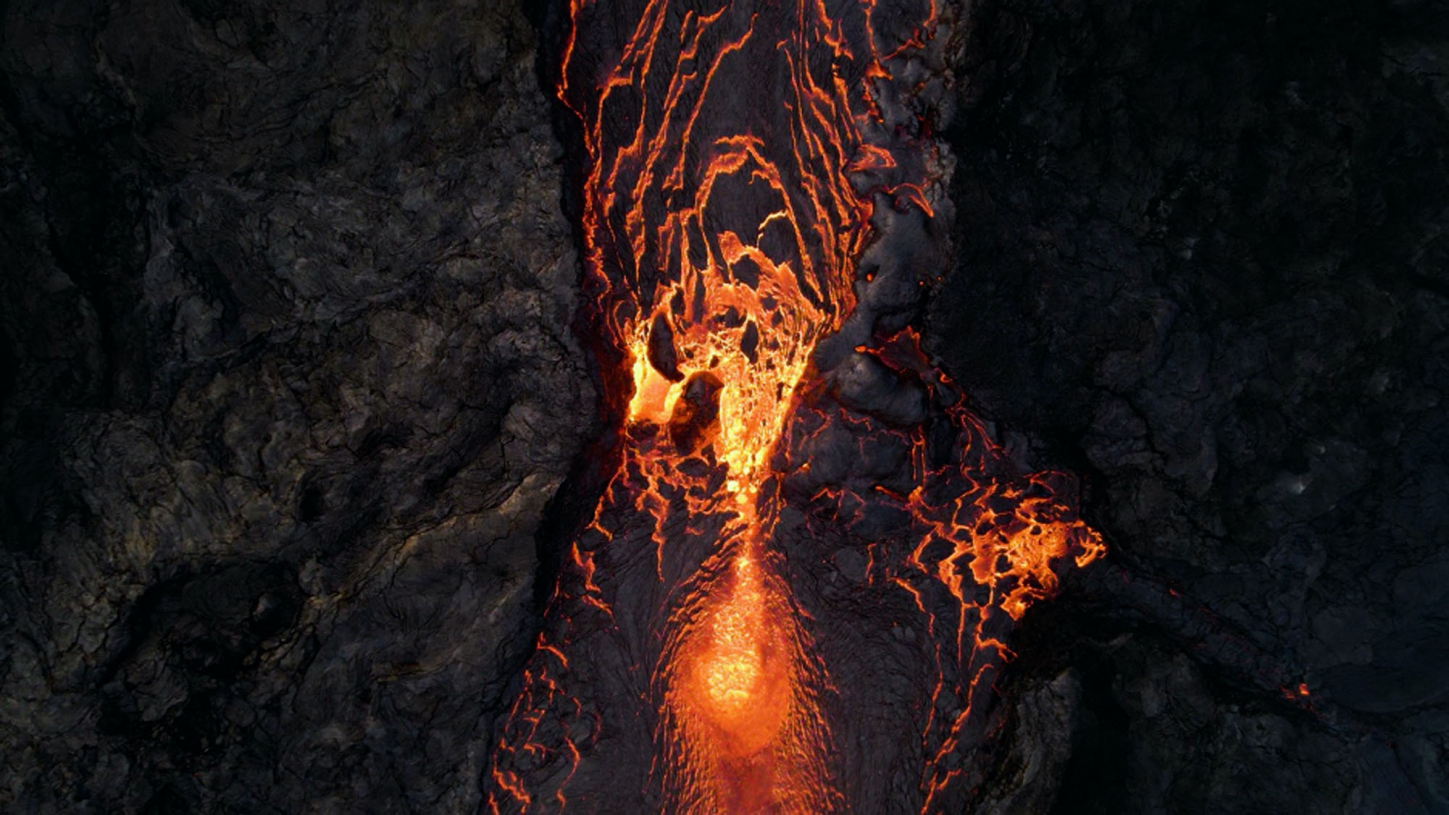
The beauty of these volcanic phenomena and the artistry of the content creation. An aerial view of an active lava channel at a pinch-point on its way over the new lava field at the Fagradalsfjall eruption, Iceland, taken on 30 May 2021. Credit: Léon Frey.

**Fig. 2:**
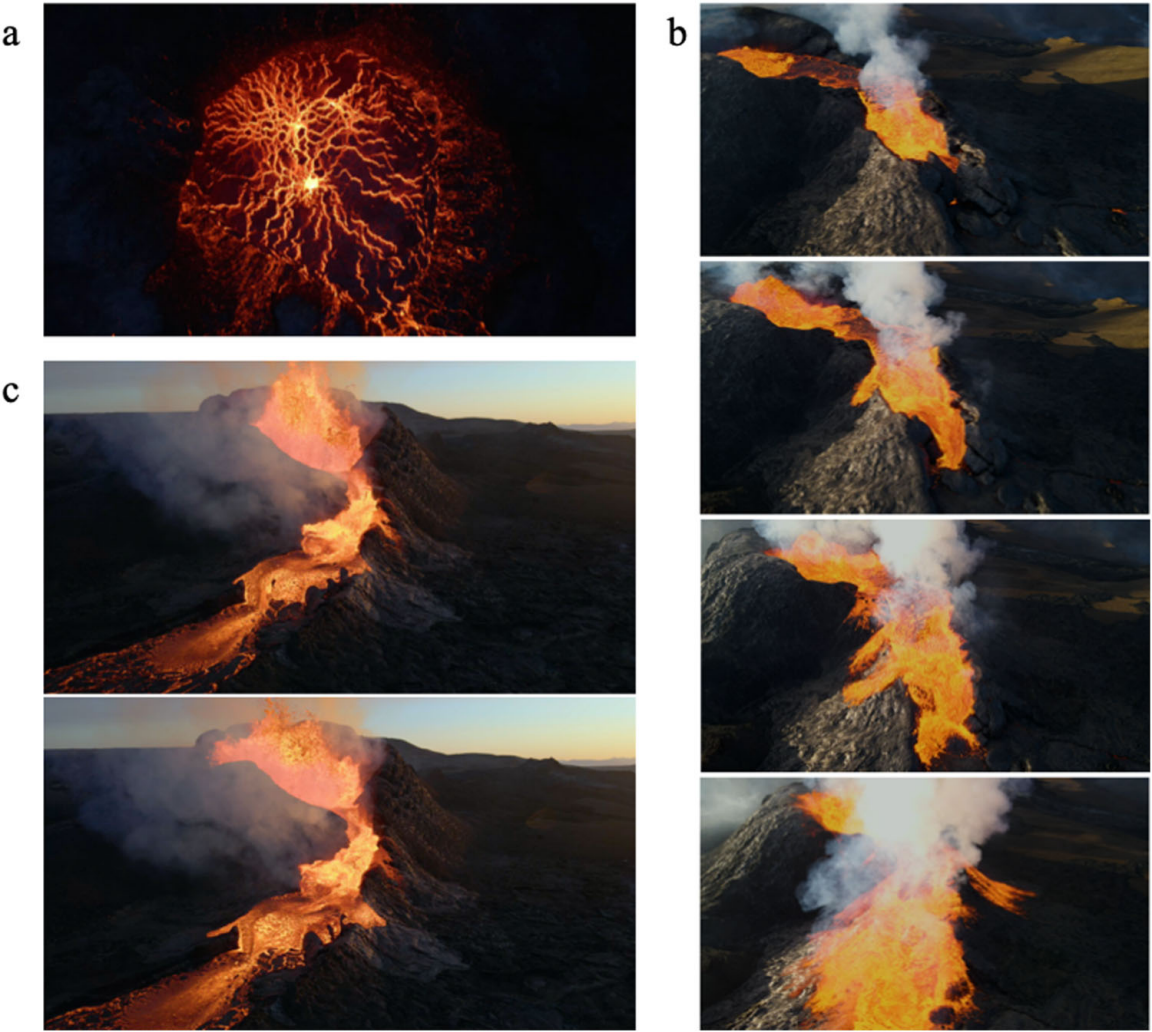
Novel observations from the Fagradalsfjall eruption, Iceland, made possible via high-resolution imagery. **a** The roiling precursors to a lava fountain in a confined vent seen from above (1 June 2021). **b** The low-fountaining height but high flux ‘lava floods’ (2 June 2021). **c** Moderate-to-high fountaining feeding lavas (22 May 2021). Credit: Léon Frey.

It is estimated that hundreds of consumer-grade drones were used by visitors to the Fagradalsfjall eruption site over the course of the eruption. As a consequence, an abundance of spectacular drone footage and ground-based imagery from multiple angles has made its way into the public domain. As one spectacular example, we reproduce a still from drone footage, showing an active lava flow against the backdrop of the new lava field (Fig. 1). Beyond the esthetics of the footage and the art of the videography, this proliferation of high-resolution imagery has already yielded surprising observations (Fig. 2).

Even without quantitative analysis, the footage permits novel interpretations of process, which could inspire new understanding of basaltic fissure eruptions. Top-down, drone’s-eye views of pseudo-stagnant ponded lava during upwelling, overflows, and transitions to fountaining have been captured in unprecedented detail (Fig. 2). These, along with similar in-crater views, provide direct confirmation of convective overturn even in localized small-volume vents^9^. The footage reveals details of the spatial extent and distribution of disturbance of the roiling pond during low-fountaining. Similarly, ‘lava floods’—short-duration, high-flux lava effusion events that overtopped the crater—have been captured in detail by both drone and ground-based videography. These floods are associated with low-fountaining, and the videography demonstrates that they result in short range lava-mantling of the vent’s cone (Fig. 2). Such qualitative observations, particularly when coupled with quantitative observations of fountain steadiness, periodicity, and height, provide new evidence to inform conceptual models of the role of decoupled gas and magma flux in controlling the dynamics of lava effusion and fountaining^10,11^.

## Cumbre Vieja

The Cumbre Vieja eruption in La Palma, Spain, was as aesthetically spectacular as Fagradalsfjall. Volcanologist and science journalist Robin George Andrews referred to the eruption in the following terms:

“…a kaleidoscope of aesthetic wonders: Incandescent ink, with hues of crimson and burnt orange, pours into the cerulean sea; streaks of purple lightning dance around skyscraper-high lava fountains; curtains of molten rock spill out of a newborn lithic coliseum, creating the youngest land on Earth.”^12^

This eruption provides an interesting counterpoint to the Fagradalsfjall eruption described above. While the Fagradalsfjall eruption was of low-intensity and mostly confined to uninhabited valleys, the Cumbre Vieja eruption demonstrated more variable, but generally greater, intensity, deposited ash and lava through bustling towns and agricultural land, and was less easy to access safely^3^. Several factors contributed to a lower number of content creators visiting and documenting the event. Formal restrictions on eruption site access and drone flight permissions, motivated by fundamental safety concerns and urban disaster-zone control, meant that access was difficult or impossible for those not involved with the emergency response or immediate scientific campaigns. Furthermore, the cost of the eruption to people’s homes and livelihoods is painfully clear around the western side of La Palma, which raises ethical concerns associated with content production for esthetic, artistic, or content-driven motivations^12^, particularly if divorced from context. The displacement of community, and the prevalence of second homes owned by expats, renders consent-seeking near impossible.

**Fig. 3:**
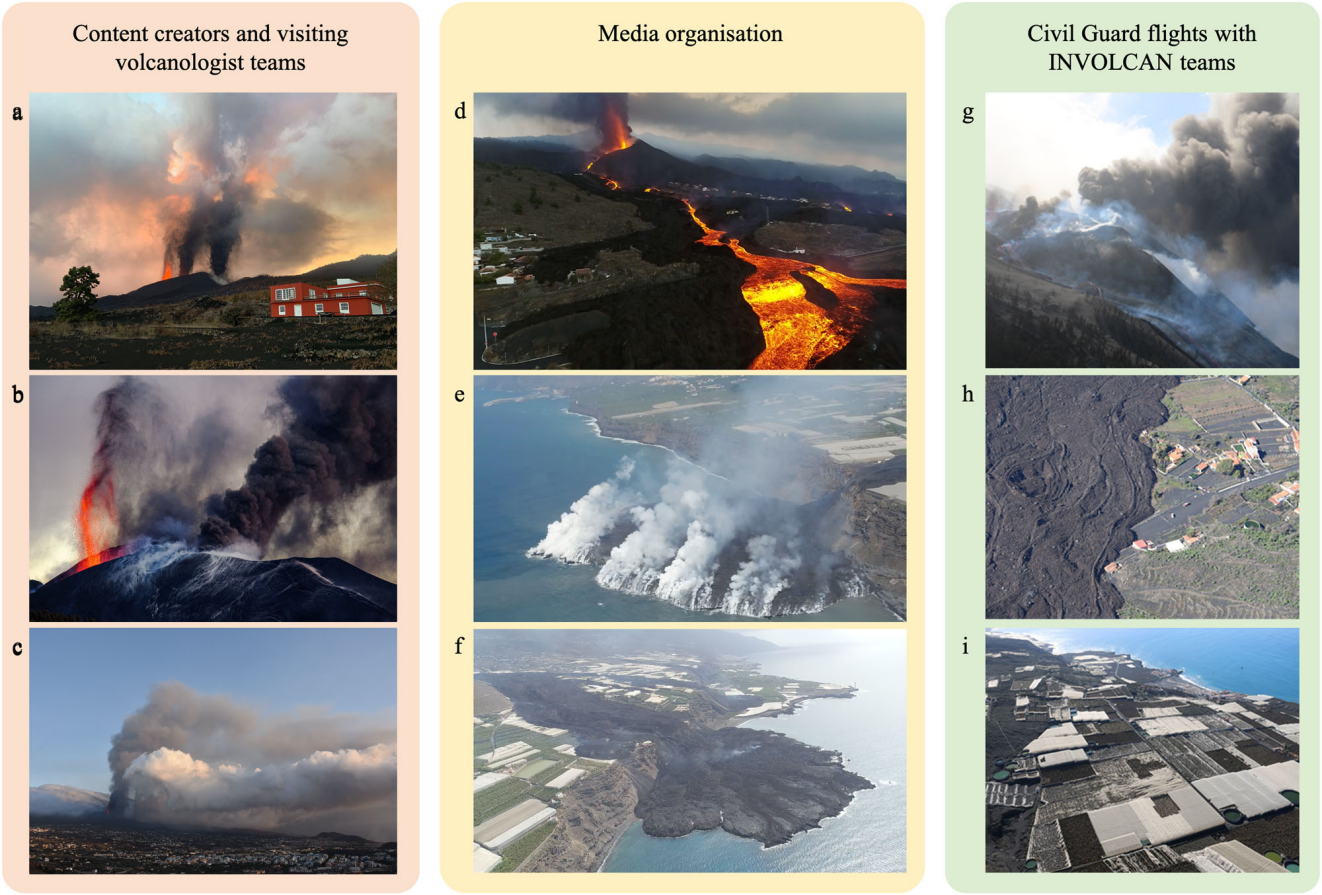
Novel observations from the Cumbre Vieja eruption, La Palma, made possible via high-resolution crowd-sourced imagery. **a** The close spatial association of different eruptive styles along-fissure localized in three vents. The upper vent (right of the vents visible in this image) manifests explosive activity and the eruption of ash and lapilli pyroclasts, while the lower vent (left of the vents visible in this image) manifests a lava fountain feeding clastogenic lavas. The middle vent between these left and right vents displays mixed eruptive style. Photograph taken on October 10^th^ 2021, credit: Ulrich Kueppers. **b** The same close spatial association of very different eruptive activity taken on December 2^nd^ 2021, credit: Juanjo Ramos. **c** Plume behavior showing the ash- and lapilli-rich dark plume separate from the ash-poor and gas-dominated lower plume, credit: Edward Llewellin. **d** A broad overview of the advancing lava system and the source fountain (looking East). **e**–**f** Repeat flights showing the evolution of the lava delta at the ocean entry point taken on **e** 30 September 2021 looking North-East, and **f** 11 November 2021 looking South-East. Credit: Alex Galdeano Rull. **g** Aerial view of the volcanic vent region taken from a helicopter piloted by the Spanish Civil Guard on 28 November 2021. **h**–**i** The encroachment of lavas on settlements and plantations. Both **h**, **i** were taken on 3 January 2022 during a helicopter flight by the Spanish Civil Guard and **g**–**i** are credit: INVOLCAN.

Nevertheless, throughout this eruption and aftermath, close access has been possible for science groups working with the emergency response teams, for media organizations, and some content creators tied to media efforts. Just as for Fagradalsfjall, the resulting videography and photography has enabled novel observations of eruption phenomenology that will influence the models that emerge for this eruption (Fig. 3). The most striking example is the simultaneous activity of different eruptive styles in close proximity to one another (Fig. 3a–c). Specifically, after the eruption onset, eruptive activity localized into discrete vents formed an uppermost (eastern-most) vent characterized by relatively high intensity explosive activity that fed an unsteady buoyant plume, and a lowermost (western-most) vent characterized by lower intensity Hawaiian fountaining that fed lavas that flowed down-slope and into the ocean. These included lava fronts supplying incandescent blocks that fell >50 m from sea cliffs, and initiated failures of cliff ledges, secondary rock falls, and related plumes of dust. At times, a middle vent between the upper and lower vents exhibited mixed activity. This close spatial association of markedly different eruptive style requires explanation in terms of the geometry of the shallow plumbing system, and the spatial organization of gas–magma decoupling processes^13,14^.

Media organizations (e.g., Overon Aerial) shared drone data with volcanologists on the ground—an explicit example of the collaborative opportunities for which we argue here—allowing researchers to target their sampling and observations of lava flow-rates and fountain heights (Fig. 3d), as well as the advance of the lava delta into the ocean (Fig. 3e, f). Dynamic hazard appraisal is safer by drone, and drone flights operated by media organizations have proven beneficial for scientists seeking access to fresh lava fronts, particularly when visible-light imaging is combined with thermal imaging. Similarly, the Civil Guard facilitated monitoring efforts by performing helicopter over-flights, footage from which could be used to assess proximal activity (Fig. 3g), damage to infrastructure and societal impacts in general (Fig. 3h, i), which can contribute to real-time hazard assessment. Direct observations from emergency services personnel were shared with science and monitoring teams actively and continuously (often via social-media and actively evolving WhatsApp groups), providing continuity when the science teams were not on-site.

## Opportunities vs the ‘volcanologist’s paradox’

Crowd-sourced and widely-shared imagery can act as direct observational inputs to conceptual model development, and as inspiration for fluid dynamic experiments designed to investigate new phenomena captured for the first time. The tourism generated by these eruptions has created a valuable scientific asset, alongside more conventional geophysical and geochemical monitoring data collected through the rapid response of volcanologists in Iceland, Spain, and worldwide.

The stream of new imagery increases the temporal resolution with which broad-scale variations in eruptions and their products can be monitored, with activity captured from multiple different angles, often simultaneously. This resource augments observational data captured by volcanology teams through field campaigns at discrete intervals^1^, and through active streaming at moderate or low resolution using webcam monitoring. Volcano observatories and partner institutions are typically under-resourced in times of eruptive crisis and response, such that crowd-sourced observational data could be an additional resource worth exploring both in near-real-time and during the post-eruption phase of prioritizing and piecing together the most incisive data. Therefore, it could be advantageous for science and monitoring teams to establish mechanisms to collect, verify and curate tourist- or media-derived imagery and observations.

While we are proposing that crowd-sourced videography—and high-resolution drone footage in particular—may be a substantial asset to volcanologists^15,16^, there are non-trivial limitations and potential drawbacks. First, as the description of the situation at the Cumbre Vieja eruption shows, there are clear safety concerns with soliciting content creators’ input at active volcanoes. Even at the lower-relative-intensity Fagradalsfjall eruption, dozens of drones are estimated to have been lost to that eruption largely due to the extremes of the environment, or unexpected changes in volcanic activity that caused non-recovery or damage to the drone^16^. Second, poorly documented footage, or footage that is not accurately time-stamped or georeferenced, is less useful, and potentially confusing. And third, a chain of users curating data sources for different purposes could increase the opportunity for data manipulation and misinformation, such as sensationalism or the exaggeration of fountain heights that require fact-checking^6^. Despite these potential pitfalls, we suggest that the opportunity presented by the increase in both resolution and quantity of eruption footage is worth exploring.

The volcanologists paradox^12^ is the tension that arises because eruptions are scientifically fascinating while also being events that have a real human cost. Recent experience has suggested that one key to reducing the tension between awe and hazard acknowledgment, is a collective sense of trust and community involvement, and ad-hoc collaboration across professional teams and local people in the field. For safety and science equally, more eyes are better than fewer, and a coordinated constellation of drones serving both purposes has merit, yet the idea is not without budgetary and feasibility challenges. One solution could involve drawing upon citizen scientists from the regional community, volcano tourists, and videographers, that could form auxiliary teams and deploy their experience to the task of observation. When conducted for both civil and scientific purposes, this would represent a whole-society task to what is, especially in the case of Cumbre Vieja, a whole-society impacting eruption.

## Social media as a meeting point for volcanologists and content creators

During the Fagradalsfjall and the Cumbre Vieja eruptions, social media has acted as a dynamic meeting point for volcano enthusiasts, photographers, videographers, and volcanologists. Platforms such as Twitter, YouTube, Facebook, and Instagram are a primary point of content exposure and sharing, and are therefore the place where the collaborations we are advocating may be born. Notably, the eruptions discussed here occurred during the global COVID-19 pandemic, which restricted the ability of many scientists and international tourists to travel to the eruption sites. These restrictions further elevated the potential value of the crowd-sourced content. For volcanologists who were not able to visit these eruption sites for logistical reasons, or due to COVID-19, the opportunity, via social media, to engage with, share, and ponder the eruptive processes has been spectacular, and has prompted vivid scientific discussion worldwide. We conclude that this is in part due to the incredible work by content creators in documenting this eruption in unprecedented detail and resolution.

## Supplementary information

Supplementary information

Supplementary Movie 1

Supplementary Movie 2

Supplementary Movie 3

Supplementary Movie 4

Supplementary Movie 5

Description of Additional Supplementary Files

## Data Availability

Data and code sharing not applicable to this article as no datasets were generated or analyzed during the current study. Raw videography used to generate the images herein is provided as Supplementary Materials.

## Code availability

Data and code sharing not applicable to this article as no datasets were generated or analysed during the current study. Raw videography used to generate the images herein is provided as Supplementary Materials.
