# Supplementary material for: Crowd-sourcing observations of volcanic eruptions during the 2021 Fagradalsfjall and Cumbre Vieja events
Source: Nat Commun. 2022 May 11;13:2611. doi: 10.1038/s41467-022-30333-4 (PMC9095650; doi:10.1038/s41467-022-30333-4)
Supplement: Supplementary file 1 — Supplementary information [file 41467_2022_30333_MOESM1_ESM.pdf]

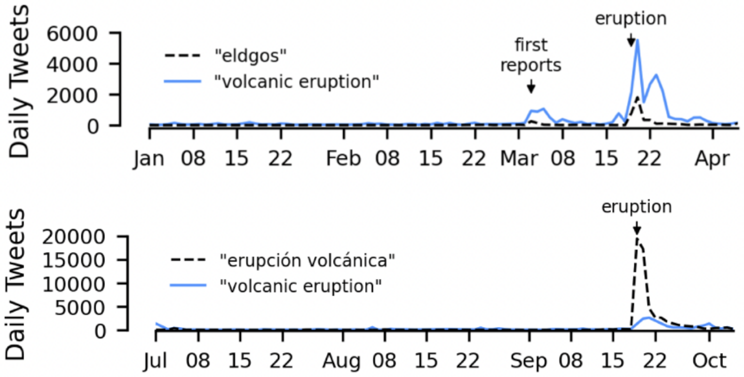

Figure S1. A time-series daily count of tweets that contain the terms (a) ‘volcanic eruption’ (English) or ‘eldgos’ (Icelandic), and (b) ‘volcanic eruption’ (English) and ‘erupción volcánica’ (Spanish). These data were crawled by Twitter API since January 2021. Tweets are binned daily. The time of the first report of volcanic unrest on the Reykjanes peninsula and the time of the eruption onsets are marked.
