# Supplementary material for: Crowd-sourcing observations of volcanic eruptions during the 2021 Fagradalsfjall and Cumbre Vieja events
Source: Nat Commun. 2022 May 11;13:2611. doi: 10.1038/s41467-022-30333-4 (PMC9095650; doi:10.1038/s41467-022-30333-4)
Supplement: Supplementary file 7 — Description of Additional Supplementary Files [file 41467_2022_30333_MOESM7_ESM.pdf]

Description of Additional Supplementary files

Filename: Supplementary Movie 1

Description: A view from a drone into a crater at the 2021 Fagradalsfjall eruption, showing the transition from lava pond convection to low-intensity fountaining. Video by Leon Frey.

Filename: Supplementary Movie 2

Description: A view from a drone of the 2021 Fagradalsfjall eruption, showing moderate intensity fountaining feeding lava flows. Video by Leon Frey.

Filename: Supplementary Movie 3

Description: A view from a drone of the 2021 Fagradalsfjall eruption, showing ‘lava flood’ phenomena documented herein. Video by Leon Frey.

Filename: Supplementary Movie 4

Description: A view from a drone of the 2021 Cumbre Vieja eruption, showing a combination of thermal (right) and optical (left) video of braided lava channels around critical infrastructure. Video by DataDron and INVOLCAN.

Filename: Supplementary Movie 5

Description: A view from a drone of the 2021 Cumbre Vieja eruption, showing a combination of thermal (right) and optical (left) video of braided lava channels around critical infrastructure including the evolution of the new lava delta. Video by DataDron and INVOLCAN.
